# Editorial: Gastrointestinal cancer immune response and immune related adverse effects

**DOI:** 10.3389/fonc.2022.1034890

**Published:** 2022-10-18

**Authors:** Ti Wen, Yanhong Deng, Bo Qin

**Affiliations:** ^1^ Department of Medical Oncology, The First Hospital of China Medical University, Shenyang, China; ^2^ Department of Medical Oncology, The Sixth Affiliated Hospital of Sun Yat-sen University, Guangzhou, China; ^3^ Department of Gastroenterology, The Sixth Affiliated Hospital of Sun Yat-sen University, Guangzhou, China; ^4^ Mayo Clinic, Rochester, MN, United States

**Keywords:** immune response, GI cancers, immune related adverse effects, tumor microenvironment, biomarker

Gastrointestinal (GI) cancers, including the cancers originating from esophagus, stomach, liver, biliary system, small intestine, colon and pancreas, (1), are among the most common and lethal solid tumors worldwide, and emerge as major health burdens, especially in China. Surgery, chemotherapy, and radiotherapy are the traditional treatments for GI cancers, but many patients have poor outcome with low 5-year survival rate (2). Recently, treatments for solid tumors targeting the crosstalk between tumor and immune system have achieved significant success in gastrointestinal cancer. Immune checkpoint inhibitor (ICI) therapy and related combination therapy have become new treatment options for gastric cancer (3), colon cancer (4), liver cancer (5), and esophageal cancer (6).

However, many questions following the application of immunotherapy raise, such as, the dynamic immune response within tumor microenvironment (TME) during cancer development and treatment. Understanding the responses of the immune system in different periods and/or changes of immune response caused by multiple treatments are necessary to reveal potential molecules as novel immune targets or biomarkers and guide personal medicine in the future.

Within this context, we proposed the research topic, aiming to publish advances in the field of the immune regulation during different cancer stages or treatment that may significantly contribute to shed light on the immunotherapy of GI cancer. After almost one year, we had received more than 50 article submissions, and 12 of them were accepted including 6 original articles, 4 review articles and 2 case reports.

## Therapeutic strategies and efficacy for GI cancers

In gastric cancer with serosal invasion, Lin et al. reported that camrelizumab combined with nab-paclitaxel plus S-1 can improve the rate of tumor regression grade (TRG 1a/1b) and pCR (pathological complete response) significantly. Duan et al. demonstrated that the combination of neoadjuvant immunotherapy and chemotherapy is correlated with high pathological and immunologic response in the tumor microenvironment (TME) of esophageal squamous cell carcinoma (ESCC). Lv et al. reported a case of advanced Epstein-Barr virus-associated gastric cancer (EBVaGC) patient, with high tumor mutation burden (TMB), positive expression of PD-L1 and PD-L1+ CD68+ macrophages enrichment, who had a long-term manageable toxicity and partial response to the combination of camrelizumab and oxaliplatin plus oral S-1 (SOX). Wang et al. reported a case of HER2-positive gallbladder cancer (GBC) patients who were resistance to trastuzumab-based targeted therapy and chemotherapy may benefit from trastuzumab plus anti-PD-1. A network meta-analysis (NMA) comparing the efficacy and safety of immunotherapy plus oxaliplatin- or cisplatin- based chemotherapy in the first-line treatment of advanced gastric cancer (AGC) was conducted by Guo et al. It suggested that the progression free survival (PFS) was prolonged significantly in patients treated with PD-1 inhibitor plus oxaliplatin- based chemotherapy. In addition, a review by Westdorp et al. discussed pathways that were altered in ICI-mediated colitis (IMC) in both human colon biopsy samples and mouse models, and revealed a complicated interplay between the gut microbiome and several types of immune cells. Thus, understanding the cellular mechanisms that induce immune related adverse events (irAEs) may provide opportunities for prevention and management.

## Identification of biomarkers in GI cancer for diagnosis and prognosis

Xu et al identified a nine-lncRNA-based signature as the ferroptosis-related prognostic model for hepatocellular carcinoma (HCC) patients. According to the prognostic signature, patients were divided into high and low risk groups, and the regulation of several immune-associated signaling pathways were correlated with the low-risk group shown by GSEA analysis. In HCC patients, Huang et al. reported that H2AFY expression was an independent unfavorable prognostic factor and correlated with immune infiltration in TME. Moreover, mitosis, cell cycle, chromatin assembly and spliceosome may be regulated by H2AFY and its co-expressed genes through E2F family and cancer-related kinases pathways shown by functional network analysis. Knockdown H2AFY inhibited the migration and proliferation of HCC cells, promoted apoptosis and cycle arrest of cells *in vitro*.

Studies by Yue et al. indicated that CX3CR1, expressed in colorectal cancer (CRC) patients and cell lines, was chosen as a TME-related hub gene. It was positively correlated with CD8+T cells, CD4+T cells, B cells, macrophages, dendritic cells and neutrophils and negatively correlated with tumor purity. Moreover, CX3CR1 expression correlated with the recruitment of immune-infiltrating cells, and it might control CRC progression through inhibiting tumor-associated macrophage (TAM) polarization. These findings suggested that CX3CR1 indicate better survival in CRC. Xie et al. reported that for CRC patient`s peripheral blood immune cells (PBIC) m^6^A RNA was a diagnostic biomarker. Compared with those in the healthy controls, the PBIC m^6^A RNA levels in the CRC group were apparently elevated, even higher in progressed and metastasized CRC, while reduced after treatment. Impressively, the area under the curve (AUC) of the PBIC m6A levels was 0.946, which was higher than the AUCs for CA125, CA19-9, and CEA. Gene set variation analysis implied that monocytes resulted as the specific immune cells most correlated with high PBIC m^6^A levels in CRC patients.

## Reviews in GI cancer progression and immunotherapy

Dugage et al. highlighted three immunotherapeutic strategies in Gastrointestinal stromal tumors (GIST). Firstly, patients involved in clinical trials must be better screened, according to the driver mutation and the tertiary lymphoid structures (TLS) or PD-L1 expression. Secondly, during imatinib therapy, indoleamine 2,3-dioxygenase (IDO) targeting should be explored after disease progression. Finally, combination of *c-kit* inhibition with ICI is recommended.

Kim and Lee. described the gut microbiome strains such as Salmonella, E. coli, F. nucleatum, B. fragilis and P. anaerobius in each stage of the tumorigenesis process of CRC. This review provided an overview of the microbiota species involved in the associations between the gut microbiome and CRC. It also indicated treatments which regulate the gut microbiome could improve the efficacy of CRC treatment.

## Conclusion

In conclusion, a group of original and review articles are collected in this Research Topic “*Gastrointestinal Cancer Immune Response and Immune Related Adverse Effects*”. We believe that this published knowledge can help us to understand the immune response in each stage of cancer development or during different types of treatment more deeply, find biomarkers and develop new therapeutic approaches for GI cancer which will contribute to improve immunotherapeutic efficacy and prognosis for GI cancer patients.

## Author contributions

All authors listed have made a substantial, direct, and intellectual contribution to the work and approved it for publication.

## Conflict of interest

The authors declare that the research was conducted in the absence of any commercial or financial relationships that could be construed as a potential conflict of interest.

## Publisher’s note

All claims expressed in this article are solely those of the authors and do not necessarily represent those of their affiliated organizations, or those of the publisher, the editors and the reviewers. Any product that may be evaluated in this article, or claim that may be made by its manufacturer, is not guaranteed or endorsed by the publisher.
